# Fish processing side streams are promising ingredients in diets for rainbow trout (*Oncorhynchus mykiss*) —Effects on growth physiology, appetite, and intestinal health

**DOI:** 10.1111/jfb.15589

**Published:** 2023-10-30

**Authors:** Niklas Warwas, Markus Langeland, Jonathan A. C. Roques, Marie Montjouridès, Jolie Smeets, Henrik Sundh, Elisabeth Jönsson, Kristina Sundell

**Affiliations:** ^1^ Department of Biological and Environmental Sciences University of Gothenburg Gothenburg Sweden; ^2^ Swedish Mariculture Research Center SWEMARC, University of Gothenburg Gothenburg Sweden; ^3^ Blue Food, Center for Future Seafood University of Gothenburg Gothenburg Sweden; ^4^ RISE Research Institute of Sweden Gothenburg Sweden

**Keywords:** alternative feed, appetite, aquaculture, intestinal health, nutrition, side stream

## Abstract

Due to the growth of aquaculture and the finite supply of fishmeal and oil, alternative marine protein and lipid sources are highly sought after. Particularly promising is the use of side streams from the fish processing industry, allowing for the recovery and retention of otherwise lost nutrients in the food production chain. The aim of the present study was to evaluate the potential of three fish processing side streams as fish feed ingredients. The side streams originated from different stages of the production chain, were used without further processing, and included sprat trimmings (heads, frames, viscera), marinated herring (fillets), and mackerel in tomato sauce (fillets and sauce). The three side streams contained moderate levels of protein (28–32% dry matter) and high levels of lipid (34–43%). The sprat trimmings included ca. 29% ash and 1.5% phosphorous, which may add value due to the high level of essential minerals but needs to be considered in feed formulations. Three diets were formulated to include 50% of each side stream replacing all fishmeal and ca. 80% of the fish oil of the control diet, which contained 35% fishmeal and 10% fish oil. The diets were evaluated in a 12‐week feeding trial using rainbow trout (*Oncorhynchus mykiss*). Fish fed the sprat diet displayed the highest feed intake and growth and showed no negative effects on the intestinal health. The mackerel side stream displayed a good digestibility but resulted in lower growth rates compared to the sprat trimmings. Fish fed the herring diet displayed the lowest performance regarding growth, feed intake, and digestibility. They further exhibited a reduction in nutrient uptake in both proximal and distal intestines, likely contributing to the observed lower digestibility and growth, and a reduction in plasma ghrelin levels. As part of a circular approach to increase marine lipid and protein production for fish feed, the tested sprat and mackerel side streams are promising raw materials; however, additional studies using more commercial‐like feed formulations are encouraged.

## 1 INTRODUCTION

Fishmeal and fish oil are central aquafeed ingredients, especially for carnivorous fish, due to their excellent amino acid and fatty acid composition (Miles & Chapman, 2006). Traditionally, capture fisheries of small pelagic fish are the primary source of fishmeal and fish oil (FAO, 2022). However, global fishery landings have been relatively stagnant for the past three decades, and it is generally agreed that the supply of fish meal and oil from wild‐caught fish will not keep pace with the growth of aquaculture and the increasing demand for feed ingredients (Miles & Chapman, 2006; Tacon & Metian, 2015). Therefore, alternative marine protein and lipid sources are highly sought after (Naylor et al., 2009). In this regard, non‐edible fish processing side streams are highly promising (FAO, 2022; Turchini et al., 2019). Utilizing such side streams to produce fish feed allows for the recovery and retention of otherwise lost nutrients in the food production system, which is critical to establishment establish of a more circular and resource resource‐efficient seafood industry (FEFAC, 2022).

Before reaching the consumer market, about 70% of fish, both from aquaculture and from capture fisheries, undergo further processing, which creates side streams (Ghaly et al., 2013). These processing side streams account for between 20% (i.e., whole fish head on gutted) and over 70% (fillet product, for instance, Thailand tilapia, Pinheiro et al., 2006) of the total fish biomass, depending on species and processing technique. They can further be divided into primary, secondary side streams, such as heads, frames, and viscera, and secondary side streams (AMEC, 2003; Ghaly et al., 2013; Guerard et al., 2002; Stevens et al., 2018). Secondary processing side streams are created along the production line due to inferior size, colouration, and texture as well through mismatches in supply and demand leading to overproduction and the loss of consumer grade products (Sandström et al., 2022). Already today, around 30% of the global fishmeal and fish oil is produced from fish processing side streams, which illustrates the high nutritional value of such side streams (FAO, 2022; Välimaa et al., 2019). Nonetheless, although the share of total fishmeal produced from side streams increases, many side streams, especially from further down the production line, remain untapped or are converted to lower‐value products such as biogas (Sandström et al., 2022). Considering their high nutritional value as well as the limited supply of fishmeal and oil, these losses are unsustainable and expensive, and studies are needed to investigate the suitability of untapped side streams as fish feed ingredients (Boyd et al., 2020; Sandström et al., 2022).

Previous studies have successfully utilized certain primary and secondary fish processing side streams in feeding trials with salmonid fish (Hedén et al., 2023; Hinchcliffe et al., 2019; Kim et al., 2019; Lee et al., 2010; Warwas et al., 2023; Ween et al., 2017). However, tailored to the respective side stream, previous studies have generally used additional processing steps to create more refined protein and lipid sources. Although needed for many side streams, additional processing steps can result in additional costs, emissions, as well as new side streams, and may not be required for certain highly nutritious side streams (Abdollahi et al., 2020; Coelho et al., 2022). Such side streams potentially include primary filleting side streams and secondary side streams such as food grade fillets rejected by the market (marinated, seasoned, and/or canned fillets). However, there is a knowledge gap regarding the direct utilization of these “raw” side streams in feeds. Fish processing side streams are highly diverse due to variations in the species of fish, processing techniques, added chemicals (such as antioxidants, preservatives, and other bioactive compounds) as well as differences in shelf life and freshness (FAO, 2022; Ucak et al., 2021). In addition, side streams from further down the production line may resemble a combination of fish and plant‐based ingredients such as canned fillets in tomato sauce. These factors may alter chemical composition, taste, microbial load, and can even introduce anti‐nutrients (Arason et al., 2009; Krogdahl et al., 2010). Therefore, each fish processing side stream must be evaluated individually and thoroughly regarding its suitability as a feed ingredient for relevant fish species.

Such an evaluation should include the nutritional composition, palatability, and digestibility of the raw material as well as potential effects on growth performance and health and welfare of the farmed fish (Glencross, 2020). In this regard, the gastrointestinal tract as the primary interface between the feed and the organism is of special interest. For example, filleting side streams are often sparsely processed and contain high levels of lipids and hemoglobin. They are therefore highly susceptible to lipid oxidation, which may affect the nutrient profile, palatability, and digestibility in salmonid fish (Hamre et al., 2001; Lund et al., 2013; Wu et al., 2022). Furthermore, additional processing steps and the inclusion of plant‐based ingredients affect the chemical composition and palatability of a side stream and can introduce anti‐nutrients, which has been shown to reduce appetite and digestibility and cause an impairment of the intestinal barrier due to inflammation (Francis et al., 2001; Knudsen et al., 2007; Knudsen et al., 2008; Krogdahl et al., 2010).

The aim of the present study was to directly utilize three locally produced fish processing side streams as feed ingredients for salmonid fish, thereby retaining their nutrients in the seafood production chain with minimal additional processing. To achieve this aim, one primary side stream (filleting trimmings from European sprat, *Sprattus sprattus*) and two secondary side streams (Atlantic herring, C*lupea harengus* in brine and canned Atlantic mackerel, *Scomber scombrus* fillets in tomato sauce) were included in diets for rainbow trout (*Oncorhynchus mykiss*) to replace all the control diet's fishmeal and most of the fish oil. The control diet contained 35% fishmeal and 10% fish oil. Three principal objectives were approached: (1) to analyze the chemical composition of the three sides streams; (2) to evaluate the effect of the experimental diets on appetite, digestibility, and feed utilization; and (3) to evaluate their effects on animal welfare and health with a special focus on the intestinal physiology.

## 2 MATERIALS AND METHODS

### 2.1 Raw materials

Three different industry side streams were used as test ingredients. These were primary filleting side streams of European sprat (*S. sprattus*, heads, frames, and viscera) and secondary side streams of Atlantic herring (*C. harengus*) fillets (removed at the second stage marination due to subpar quality) and canned Atlantic mackerel (*S. scombrus*) in tomato sauce (a distribution and retail side stream). All the raw materials were kindly provided by Renahav Sverige AB, a biogas and fertilizer producer in Kungshamn, Sweden. The sprat trimmings were collected during the filleting process, and salt (5% NaCl) was added for preservation. The marinated herring fillets were collected from their marination barrels, washed using tap water to remove excess brine, and drained using a sieve (2 mm mesh). Similarly, 600 cans containing mackerel fillets and tomato sauce were opened manually and washed as described earlier. All the three products were dried (forced air, Cewatech AB, Gothenburg & Nordic Paper Seffle AB, Säffle, Sweden) and milled through a 1 mm screen (Novital Davide, Novital S.r.l., Lonate, Pozzolo, Italy). The chemical composition of the test ingredients is presented in Table 1. A more detailed chemical composition, including the amino acid and fatty acid profiles of the three raw materials, is presented in Table A1 in Data S1. However, due to limited quantities of the raw material for the feed production, the analyses in Table A1 in Data S1 were performed on different batches of the raw materials that were not washed, dried, or ground as described earlier.

**TABLE 1:** Proximate (g/100 g DM), gross energy (MJ/kg DM), and mineral (g/kg DM) composition of the washed, dried, and ground test ingredients: mackerel in tomato sauce, marinated herring, and sprat trimmings.

	Mackerel	Herring	Sprat
Dry matter (DM, %)	88.4	69.6	89.1
Ash	7.8	22.7	29.4
Crude protein^a^	29.8	32.1	28.1
Crude lipid^b^	34.9	43.0	34.8
Gross energy^c^	21.9	22.7	20.1
Calcium	0.6	0.2	23.7
Potassium	15.7	0.9	2.4
Magnesium	1.0	0.1	0.9
Sodium	11.9	52.6	62.0
Phosphorous	3.3	1.2	14.7
Selene	2.8	2.4	2.6

^a^ According to Kjeldahl (N × 6.25).

^b^ According to Schmid‐Bondzynski‐Ratslaff.

^c^ Analysed using oxygen bomb calorimetry.

### 2.2 Experimental diets

Four isocaloric and isonitrogenous diets were formulated to fulfill the nutrient requirements of rainbow trout (Hua & Bureau, 2019; NRC, 2011), and produced at the Feed Technology Laboratory, Swedish University of Agricultural Sciences (SLU), Uppsala, Sweden. The control diet contained conventional feed ingredients, including 35% fishmeal and 10% fish oil (Table 2). The three experimental diets included 50% of the test ingredient, respectively, and replaced 100% of the fishmeal. Due to the higher protein content in commercial fishmeal compared to the three side streams, wheat meal and wheat gluten were used to balance the diets. Due to the high lipid content of the side streams, most of the fish oil (between 76% and 86%) and all of the vegetable oil present in the control diet were replaced in the experimental diets. The high inclusion level, 50%, was chosen to be able to replace all the fishmeal and most of the fish oil to challenge the fish and to screen for any possible physiological as well as health and welfare effects in response to the test ingredients. All three alternative raw materials were fish based and contained high amounts of essential amino and fatty acids (Table A1 in Data S1); thus a 50% inclusion level was feasible from a nutritional perspective (Hua & Bureau, 2019). As the aim of the present study was to screen possible physiological effects of the three side streams, the diets were formulated to include as high levels as possible rather than to mimic commercial diets. This led to lower total lipid (17%–19%) and energy levels (20 MJ/kg) in all four experimental diets when compared to commercial diets (25%–35% and 24 MJ/kg, respectively). For the feed production, the ingredients were mixed in a horizontal drum mixer before gelatin and hot water (ca. 50°C) were added. The diet mash was then pressed through a single mesh meat grinder (hole plate 3 mm, Nima Maskinteknik AB, Örebro, Sweden) and heated in a stream oven to induce gelatinization of the starch (103°C Electrolux, 6 GN1/1, Stadshagen, Sweden). Diet strings were air‐dried for 18 h and cut into pellets using a blender (Kneubühler, Luzern, Switzerland). The diets were then transported to the Department of Biological and Environmental Sciences (BioEnv), Gothenburg University (GU) and stored at 4°C until use further in the feeding trial.

**TABLE 2:** Diet formulation (g/100 g), proximate composition (g/100 g) and energy content (MJ/kg) of the experimental diets: control (C), mackerel (M), herring (H) and sprat (S).

	C	M	H	S
Ingredient				
Fishmeal^a^	35.0	–	–	–
Soy protein concentrate^b^	20.0	20.0	20.0	20.0
Wheat gluten^c^	–	16.0	17.0	14.4
Wheat meal	15.0	–	–	6.0
Gelatin	6.0	6.0	6.0	6.0
Fish oil^d^	10.0	2.4	2.0	1.4
Rapeseed oil	3.8	–	–	–
Vitamin mineral premix	1.7	1.7	1.7	1.7
Cellulose	8.0	–	–	–
Dried mackerel in tomato sauce	–	50.0	–	–
Dried herring filet	–	–	50.0	–
Dried sprat trimmings	–	–	–	50.0
Titanium dioxide	0.5	0.5	0.5	0.5
Potato starch	–	3.4	4.8	–
Proximate composition				
Dry matter	93.8	92.6	90.8	93.57
Ash	8.1	5.4	11.6	15.58
Protein^e^	43.3	44.0	45.0	42.60
Fibre (85°C)	6.6	1.9	1.8	1.21
Crude lipid^f^	17.5	18.8	18.8	17.09
Gross energy^g^	21.03	21.45	20.96	19.69

^a^ Low‐temperature fish meal, North Atlantic origin.

^b^ Hamlet, Horsens, Denmark.

^c^ Lantmännen Reppe, Lidköping, Sweden.

^d^ North Atlantic origin.

^e^ According to Kjeldahl (N × 6.25).

^f^ According to Schmid‐Bondzynski‐Ratslaff.

^g^ Analysed using oxygen bomb calorimetry.

### 2.3 Chemical analysis

Both the side streams and diets were analysed for their chemical composition. Dry matter (DM) content was determined by weighing the sample before and after drying at 103°C for 16 h followed by cooling in a desiccator. The ash content was measured by heating samples to 550°C for 24 h until the weight of the sample was stable and the ash was completely white. Samples were then cooled in a desiccator and weighed (AOAC 1995). The crude protein (CP) content was estimated from the total nitrogen content (N x 6.25, Nordic Committee on Feed Analysis, 1976). Total nitrogen was analysed according to the Kjeldahl method using a 2020 digester and a 2400 Kjeltec Analyzer unit (FOSS Analytical A/S, Hillerød, Denmark). The crude lipid (CL) content was determined using hydrolyzation (1047 Hydrolysing Unit, Soxtec System HT 1043 Extraction Unit; FOSS Analytical A/S, Hillerød, Denmark). The neutral detergent fibre (NDF) determination was carried out according to Mertens (2002). Isoperibol bomb calorimetry was carried out to determine gross energy (GE, Parr 6300, Parr Instrument Company, Moline, IL, USA). Amino acids and fatty acids were analysed at a certified laboratory (Eurofins Food & Agro Testing Sweden AB, Linköping, Sweden). Amino acids were analysed using HPLC. Fatty acids were determined using gas chromatography after conversion to fatty acid methyl esters.

### 2.4 Feeding trial

The feeding trial was carried out in a 10°C freshwater recirculating aquaculture system (FW‐RAS) at BioEnv, GU. Juvenile rainbow trout (ca. 50 g) were purchased from Vänneåns Fiskodling AB (Knäred, Sweden) in March 2020. The fish were housed in cylindrical fibreglass tanks (80 L, flow rate ca. 1 L/min) covered with half opaque and half transparent plexiglass for partial shading. The light regime was set to 12 h light and dark, and the fish were acclimatized to the experimental conditions for 4 weeks. Two weeks before the start of the experiment, the fish were anesthetized (MS‐222, Finquel, Argent Chemical Laboratories, Redmond, WA, USA, 0.08 g/L), and passive transponder tags (12 mm, Biomark, Boise, ID, USA) were inserted into the peritoneal cavity via a small incision laterally between the pectoral and pelvic fins, using a sterile scalpel. At the start of the feeding trial, the fish were anesthetized as described earlier, measured for weight and length and distributed among 12 experimental tanks (the same system as described earlier), 3 tanks per treatment with 15 fish per tank (62.6 ± 0.4 g). The feeding trial was carried out over 12 weeks (April – July 2020). The fish were fed by hand twice daily, 7 days per week at 9:00 a.m. and 15:00 p.m. Uneaten pellets were collected using a sieve (0.5 mm mesh) 20 min after feeding at the tank outlet after lifting the standpipe, oven‐dried overnight at 37°C, and weighed to determine the daily feed intake (*FI*, Equation 3). The drying process for each diet was validated using a recovery test by submerging 100 g of feed in the FW‐RAS water for 5 h before the pellets were collected and oven‐dried as described earlier. The feed ration was set to 1.5% body weight per day (BW/d) and increased in increments of 10% (i.e. from 1.5% to 1.65% to 1.815%) weekly or earlier if feed waste was below 5% for two consecutive days. Fish were weighed every 4 weeks (including at the final sampling), and the feed ration was readjusted to 1.5%.

Animal care and all experimental procedures were performed in accordance with the guidelines and regulations set by the ethical permit (244–2018, diarienummer: 5.8.18–15096/2018) under Swedish and EU‐legislation and approved by the ethical committee on animal research in Gothenburg, Sweden. All fish were inspected daily for loss of equilibrium, skin and fin damage, other physical impairments, and general abnormal behavior according to the endpoint description set out in the ethical permit.

### 2.5 Sampling

At the end of the feeding trial, tissue and blood sampling was carried out over three consecutive days with two sampling events per day (two tanks per sampling event), resulting in a total of six sampling events. This sampling schedule was chosen to enable in vitro intestinal measurements using the Ussing chamber method (see below). The fish were fed according to schedule up until the day before the respective sampling. At each sampling, four individuals were randomly netted from each of two tanks and killed using an anesthetic overdose (10 mg/L metomidate hydrochloride, Aquacalm, Syndel, Canada) followed by a sharp blow to the head. Blood samples were taken from the caudal vessels using a heparinized syringe (1 mL), and hematocrit (Hct, %) and hemoglobin (Hb, g/dL) were analysed immediately. The remaining blood was centrifuged (Thermo Scientific Heraeus Pico 17, Thermo Fisher Scientific, Waltham, MA, USA) to separate blood cells from plasma. The plasma was transferred into separate tubes and stored at −80°C until further analysis. The muscle fat content of whole fish was measured carried out using the a Distell fish fat meter (Distell Inc., West Lothian, Scotland).

The intraperitoneal cavity was opened, and the intestine, between the last pyloric ceca and the rectum, was removed using blunt dissection. The intestines was divided at the ileorectal valve into two sections, the proximal intestine and distal intestine. Each intestinal region was further divided into two differently sized sections for two separate analyses. For histology, 2 mm of the most anterior part of each region was transferred to buffered formaldehyde (4%) for 24 h and thereafter stored in 70% ethanol until further analysis. For the electrophysiological analysis, the intestine was opened longitudinally, and ca. 2 cm of both proximal intestine and distal intestine were carefully, cleaned to remove fat and feces, transferred to ice‐cold Ringer's solution (Ringer solution for FW salmonids according to Sundell et al. (2003), with the addition of 0.5 mM lysine), and kept on ice for ca. 20 min before they were mounted into the Ussing chambers. Liver and viscera were excised and weighed. To remove the hypothalamus, fish were decapitated and the brain was removed. The hypothalamus was dissected using fine forceps and a scalpel, wrapped in aluminum foil, snap frozen in liquid nitrogen, and stored at −80°C for gene expression analysis.

### 2.6 Calculations

Weight gain (*WG*) was calculated by subtracting the initial body weight (*IBW*) from the final body weight (*FBW*). *IBW, FBW*, and the experimental period in days (*d*) were used to calculate the specific growth rate (*SGR*) according to Equation 1. The condition factor (*CF*) was calculated using the fork length (*L*) and *FBW* (Equation 2). The average feed intake per fish (*FI*) was calculated by subtracting recovered (*F*_*out*_) from given feed (*F*_*in*_) divided by the number of fish per tank (*n*, Equation 3). The feed conversion ratio (*FCR*) was calculated using the dry weight of the consumed feed (*FI*) and the *WG* (Equation 4). Using liver and viscera weight, the hepato‐somatic (*HSI*, Equation 5) and viscerosomatic index (*VSI*, Equation 6) were calculated. Survival was expressed in percentage using the initial number of individuals (*n*_*i*_) and the number of individuals at the final sampling day (*n*_*f*_, Equation 7). Using the concentration (0.5%) of the inert marker titanium dioxide (TiO2, *I*) and the relative concentration of nutrients (*N*) in diet (*D*) and feces (*F*) the apparent digestibility coefficient (*ADC*) of the diets was calculated according to Cho et al. (1982); Equation 8).

(1) $$\mathrm{SGR}\;\left(\%\mathrm{BW}/\mathrm{day}\right)=\left(\ln\;\mathrm{FBW}-\ln\;\mathrm{IBW}\right)/d\times 100$$

(2) $$CF=\left(\mathrm{FBW}/L^{3}\right)\times 100$$

(3) $$FI\;\left(g/fish\right)=\left(F_{\text{out}}‐F_{in}\right)/n$$

(4) FCR=FI/WG

(5) $$\mathrm{HSI}\;\left(\%\right)=\text{liver weight}/\mathrm{FBW}\times 100$$

(6) $$\mathrm{VSI}\;\left(\%\right)=\text{viscera weight}/\mathrm{FBW}\times 100$$

(7) $$\text{Survival}\;\left(\%\right)=n_{f}/n_{i}\times 100$$

(8) $$\mathrm{ADC}\;\left(\%\right)=100-\left(\mathrm{ID}\times \mathrm{NF}\right)/\left(\text{IF}\times \mathrm{ND}\right)\times 100$$

### 2.7 Blood and plasma parameters

For *Hct*, 80 μL of duplicate samples was drawn into capillary tubes and centrifuged (Haematokrit 210, Hettich, Tuttlingen, Germany) at 10,000 rcf for 5 min. A Hawksley reader was used to determine *Hct*. For *Hb*, a handheld Hb 201+ meter was used (Hemocue AB, Ängelholm, Sweden), and the values were corrected for fish blood as determined by Clark et al. (2008). Using Hct and Hb, the mean corpuscular hemoglobin content (*MCHC*, Equation 9) was calculated.

(9) MCHC=Hb/Hct×10

Plasma cortisol levels were analysed using a radioimmunoassay as described by Young (1986) using a cortisol antibody (code: S020; lot: 1014–180,182, Guildford Ltd., Guildford, Surrey, UK, validated by Sundh et al., 2011) and tracer (hydrocortisone‐[1,2,6,7‐3H(N)], NEN Life Sciences Products, Boston, MA, USA). Concentrations were determined using a standard curve of hydrocortisone (Sigma, St. Louis, MO, USA). Radioactivity was measured after addition of 4 mL Ultima Gold (PerkinElmer, Waltham, MA, USA) using a beta counter (Wallac 1409, LKB Instruments, Turku, Finland). Plasma ghrelin levels were analysed using a radioimmunoassay as described by Jönsson et al. (2007), modified from Hosoda et al. (2000), using an antibody kindly provided by Dr. Hiroshi Hosoda (Osaka, Japan, see Hosoda et al., 2000). Human ^125^I‐ghrelin (Perkin Elmer) was used as tracer. Radioactivity was measured using a gamma counter (Wallac 1417, LKB Instruments). The glucose concentration was determined using an enzymatic assay kit (GHK, Sigma, St. Louis, MO, USA). A cryoscopy osmometer was used to analyse plasma osmolarity (Advanced Model 3320 Micro‐Osmometer, Advanced Instruments Inc., Norwood, MA, USA).

### 2.8 Intestinal barrier function and health

In vitro measurements of intestinal health were carried out using the Ussing chamber technique as described in detail by Sundell and Sundh (2012). In short, the serosal layer of each intestinal section was peeled off using surgical forceps under a stereomicroscope. The tissues were then mounted in Ussing chambers (area of exposure: 0.75 cm^2^) and 4 mL of the Ringer's solution was added to both sides of the tissue (serosal and mucosal). A gas lift driven by a gas mixture of air and 0.3% CO_2_ was used to mix the Ringer's solutions, aerate the tissue, and maintain a pH of 7.8. The temperature in the chambers was set at 10°C using a water‐supplied cooling mantle. The electrochemical parameters transepithelial resistance (TER), transepithelial potential (TEP), and the short‐circuit current (SCC) were measured at 5‐min intervals over 150 min. Averages of the last 20 min were used for the statistical comparisons. For intestinal epithelia of fish, TER reflects the paracellular resistance, TEP reflects the potential difference between serosal and mucosal sides generated by active and passive ion fluxes, and SCC reflects the currents generated across the epithelium by active transport processes (Sundell & Sundh, 2012). After 60 min, when the tissues had reached steady state, the Ringer's solution was replaced with a fresh Ringer's solution, and two radiolabeled marker molecules were added to the mucosal side. ^14^C‐mannitol (1.24 × 10^14^ dpm/mol, Moravek Biochemicals, Brea, CA, USA), an inert, small, and hydrophilic sugar molecule was added to assess the apparent paracellular permeability and epithelial integrity. ^3^H‐Lysine (7.1 × 10^16^ dpm/mol, Moravek Biochemicals), an essential amino acid and often a limiting amino acid in aquafeeds, was used to quantify active amino acid uptake kinetics. Aliquots of 50 μL were taken at minute 0 from both serosal and mucosal side and at minutes 20, 25, 30, 60, 80, 85, and 90 from the serosal side of the chamber and analysed for ^3^H and ^14^C using a dual label protocol and a beta counter (Wallac 1409, LKB Instruments, Turku, Finland). The apparent permeability of mannitol (*Papp*, Equation 10) was calculated using the appearance rate of ^14^‐C‐mannitol (*dQ/dT*), the area of expoure (*A*) and the initial concentration of ^14^C‐ mannitol on the mucosal side (Equation 11). ^3^H‐lysine transport was calculated using the appearance rate of labeled lysine on the mucosal chamber side (*dQ/dT*) and the area of exposure (A).

(10) $$\text{Papp}\;\left(\mathrm{cm}/s\right)=\mathrm{dQ}/\mathrm{dT}\times 1/A\times C_{0}$$

(11) $$\text{Lysine}\;\left(\mathrm{mol}\times \min^{-1}\times {\mathrm{cm}}^{-2}\right)=\mathrm{dQ}/\mathrm{dT}\times 1/A$$

### 2.9 Histology

The intestinal samples were dehydrated using a standard ethanol gradient followed by Histolab Clear (Histolab Products AB, Askim, Sweden). The dehydrated samples were embedded in paraffin using a tissue processor (TP 1020, Leica, Wetzlar, Germany). From each tissue, six 5 μm thick nonconsecutive cross‐sections were produced (Shandon Scientific; Labex Instrument, Helsingborg, Sweden) and mounted on slides coated with 3′‐aminopropyltriethoxysilane (APES; Merck KGaA, Darmstadt, Germany). The sections were rehydrated and stained with hematoxylin (Histolab Products AB, Askim, Sweden), eosin (Histolab Products AB), and alcian blue 8 GX (pH 2.5, Merck, KGaA). Two pictures of each section were taken using a 2.3 MP camera (PowerPack ace 2.3 MP, Basler AG, Ahrensburg, Germany) connected to a microscope (Eclipse E1000, Nikon, Tokyo, Japan, x10 magnification). The resulting 12 images per tissue were analysed for villi length, lamina propria width, submucosa width, goblet cell count and vacuolization using the ImageJ software (Wayne Rasband, NIH, USA). The supranuclear vacuolization was scored according to Knudsen et al. (2007). Six fish were analysed for each dietary treatment.

### 2.10 Gene expression

Hypothalamic RNA was extracted using the RNeasy Plus Mini Kit (Qiagen NV, Hilden, Germany). Tissues (20–30 mg) were homogenized in 2 mL tubes containing 600 μL lysis buffer (RLT plus buffer) and a stainless‐steel bead using the TissueLyser II (Qiagen NV). The RNA concentration and purity were determined using ultraviolet spectrophotometry at 260 nm (NanodropTM One/OneC, Thermo Fischer Scientific). In addition, the RNA quality of 24 samples, including 16 samples with low RNA concentrations (<50 ng/μL), was evaluated using the RNA Integrity Number (RINe TapeStation Agilent Technologies 2200, Santa Clara, CA, USA). To generate cDNA the iScript cDNA Synthesis Kit (Bio‐Rad Laboratories Inc., Richmond, CA, USA) and DEPC‐treated water (Thermo Fischer Scentific, Waltham, MA, USA) were used. Samples with RNA concentrations <20 ng/μL were excluded from the analysis.

Relative gene expression was determined by real‐time quantitative PCR (RT‐qPCR). RT‐qPCR reactions were carried out in duplicates using a primer concentration of 0.5 μM and the SsoAdvanced Universal SYBR Green Supermix (Bio‐Rad Laboratories Inc.). Primer pairs of target and reference genes are presented in Table 3. Primer efficiency was confirmed using a dilution series of cDNA (0.78–25 ng) from six randomly pooled samples (efficiency range of 95%–110%). The RT‐qPCR reaction was carried out over 40 cycles with 95°C denaturation and 60°C annealing and extension (CFX96 Connect Real‐time PCR Detection System, Bio‐Rad Laboratories Inc.). To verify product purity, melting curves were analysed for all samples. To confirm product size for each primer pair, agarose gel electrophoreses (1.2% agarose in TAE buffer, ran at 90 V for 1 h) was carried out.

**TABLE 3:** Nucleotide sequence of primers used to evaluate mRNA concentration of target genes that regulate appetite in the hypothalamus of juvenile rainbow trout.

Gene	Direction	Sequence	Accession number	Reference
CART	F	GTCCATCGTTCTTAGTGCTGAA	AB455538	Jørgensen et al., 2016
	R	CAG TTGCTTTTCGTT GGTCAA		
CRF	F	ACAACGACTCAACTGAAGATCTCG	NM001124286	Jørgensen et al., 2016
	R	AGGAAATTGAGCTTCATGTCAGG		
MC4R	F	TTCTCACACTGGGGATAGTCA	AY534915.1	Jørgensen et al., 2016
	R	CACAGCCAAAGAACAGATGAAT		
NPY	F	AGAATTGCTGCTGAAGGAGAG	AF203902	Jørgensen et al., 2016
	R	GGGACAGACACTATTACCACAA		
POMCA1	F	CTCGCTGTCAAGACCTCAACTCT	TC86162	Leder and Silverstein, 2006
	R	CAATAACCACGCAGGACACA		
Ubiquitin	F	AGATAAATCGGAGAGTTGCTGTG	X99303	Carney Almroth et al., 2008
	R	CCTGCTCCACCTTGTGTTGT		
β‐Actin	F	ATGGAAGATGAAATCGCC	AF157514	–
	R	TGCCAGATCTTCTCCATG		

Abbreviations: CART, cocaine‐ and amphetamine‐regulated; CRF, corticotropin‐releasing factor; F, forward; MC4R, melanocortin 4 receptor; NPY, neuropeptide Y; POMCA1, proopiomelanocortin A1; R, reverse.

Relative gene expression of the target genes was calculated using the 2 − ΔCT’ method (Equation 12).

(12) $$\text{Relative expression}=2_{T}^{-\left(C_{T}\left(\text{reference}\right)-C_{T}\left(\text{target}\right)\right)}$$

### 2.11 Statistical analysis

The statistical analyses were performed using SPSS 29 (SPSS Inc., Chicago, IL, USA). Normality was tested using a Shapiro–Wilk test and visual inspection of the residual Q‐Q plots. Homogeneity of variances was tested using Levene's test. The data were analysed using a nested ANOVA with the two fixed factors, tank and treatment, where tank is nested within treatment. The number of replicates (*n*) is reported per tank. Effects on intestinal histology were analysed using a one‐way ANOVA. Pairwise comparisons were performed using Tukey's post‐hoc test adjusted for multiple comparisons. When normality or homogeneity was violated, the data set was transformed using a log10 transformation to meet the requirements, or, if unsuccessful, a nonparametric Kruskal‐Wallis followed by Dunn's test was performed. Significance was assumed at *p* < 0.05. Values are presented as means ± SD.

## 3 RESULTS

### 3.1 Raw materials and diets

The chemical composition of the raw materials is presented in Table 1. All the ingredients were high in lipid (35%–43%). The highest lipid levels were observed for the herring side stream. The protein content of the test ingredients was moderate with the highest values for the herring fillets (32%). The highest level of phosphorous was observed in the sprat product with 1.47% and the lowest for herring with 0.12%. Additionally, both the herring and sprat products contained high amounts of ash (22% and 29%, respectively) mainly due to NaCl (Table 1).

### 3.2 Growth and feed utilization

**FIGURE 1:**
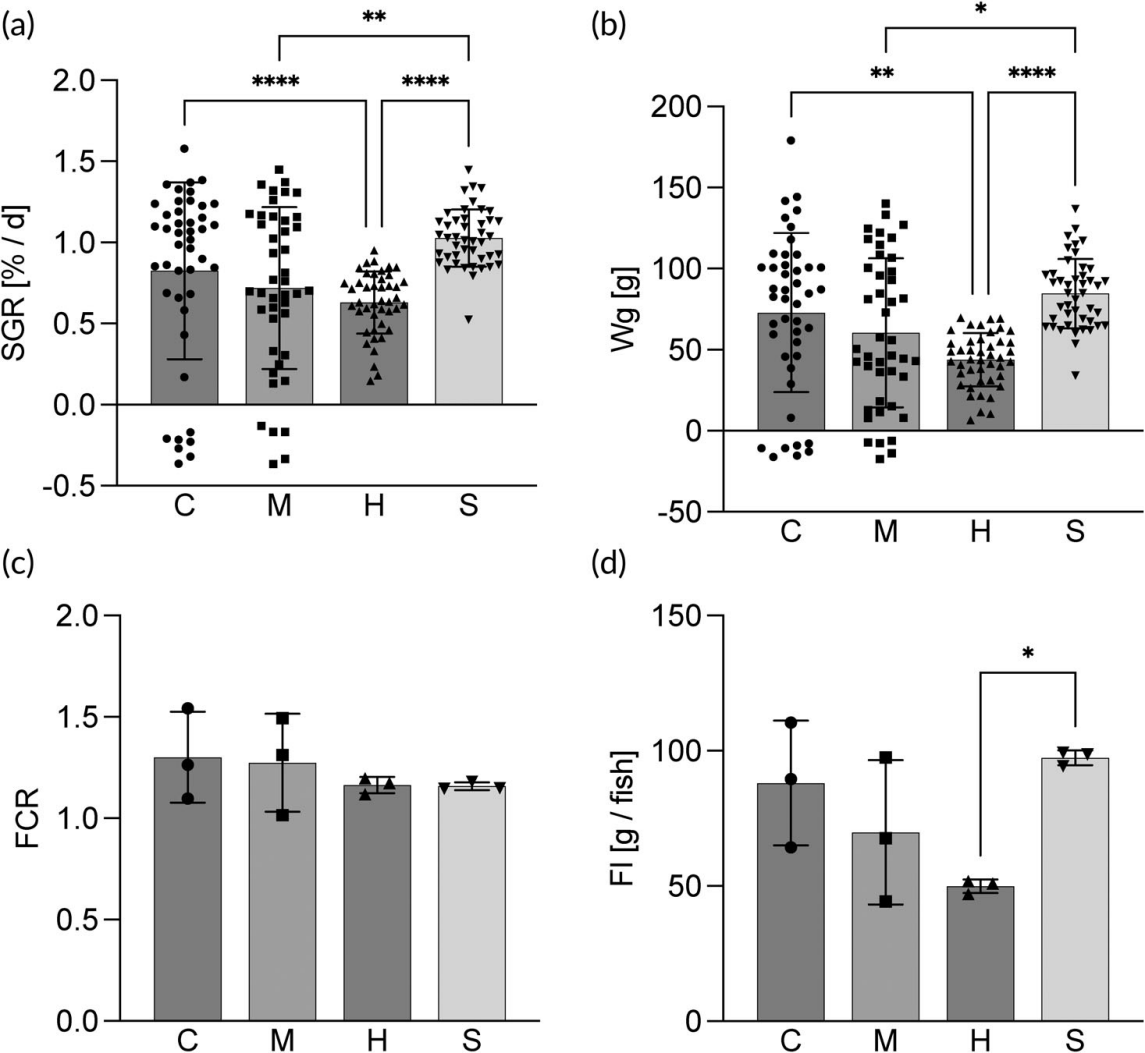
Growth parameters and body indices of rainbow trout fed four different experimental diets, control (C), mackerel (M), herring (H), and sprat (S). (a) Specific growth rate (SGR), (b) weight gain (Wg), (c) feed conversion ratio (FCR), and (d) feed intake (FI). Bars indicate mean ± SD. Individual data points are individual fish for SGR and WG, and replicate tanks for FCR and FI. Symbols refer to significant differences between diets, *p* < 0.0001 (****); *p* < 0.001 (***); *p* < 0.01(**); and *p* < 0.05 (*).

The growth of the fish was significantly affected by the experimental diets (Figure 1). The highest WG and SGR were observed for the control (73 ± 49 g and 0.82 ± 0.5% respectively) and sprat diets (85 ± 21 g and 1.03 ± 0.2% respectively). Both exhibited higher SGRs than the herring treatment (0.63 ± 0.2%). Additionally, the SGR of the fish fed sprat diet was higher than that of the fish fed the mackerel diet (0.72 ± 0.5%). Although FCR was unaffected by diet, the FI varied significantly between the treatments with the highest values for fish given the sprat diet (97.5 ± 2.7 g/fish) and significantly lower values for the herring diet (49.9 ± 2.5 g/fish). Fish fed the control and mackerel diets displayed intermediate FI values of 88.1 ± 23.1 and 69.9 ± 26.7 g/fish respectively (Figure 1). No differences were observed between the dietary treatments for HSI, VSI, CF or survival (Table 4).

**TABLE 4:** Growth parameters and body indices of rainbow trout fed one of the experimental diets; control (C), mackerel (M), herring (H) and sprat (S).

	Diet				*p*‐value	
	C	M	H	S	Diet	Tank
Initial weight, g (*n* = 15)	60.7 ± 5.2	59.9 ± 5.4	60.0 ± 5.2	59.7 ± 5.0	0.812	0.999
Final weight, g (*n* = 15)	133.7 ± 50.0^ab^	120.5 ± 47.1^bc^	103.6 ± 18.7^c^	144.3 ± 23.0^a^	<0.001^1^	
Condition factor (*n* = 15)	1.28 ± 0.21	1.28 ± 0.22	1.29 ± 0.18	1.27 ± 0.14	0.590^1^	
HSI (*n* = 4)	1.46 ± 0,30	1.44 ± 0.32	1.66 ± 0.24	1,46 ± 0.16	0.127	0.302
VSI (*n* = 4)	12.90 ± 4.17	12.97 ± 4,37	13.84 ± 1.50	13.84 ± 1.50	0.240^1^	
Fat (*n* = 4)	2.43 ± 0.53	2.63 ± 1.03	2.16 ± 0.53	2.72 ± 0.46	0.110	0.013
Survival, % (*n* = 3)	97.78 ± 3.85	95.56 ± 3.85	97.78 ± 3.85	97.78 ± 3.85	0.859	

*Note*: The data are shown as mean ± SD. Values within rows with different superscripts are significantly different (*p* < 0.05).

Abbreviations: HSI, hepato‐somatic index; VSI, viscerosomatic index.

^1^ *p*‐values obtained from a non‐parametric Kruskal‐Wallis analysis of variance.

Twelve fish lost weight during the experimental period. These fish were all found in four tanks, two with control fish (7 fish) and two with fish fed the mackerel diet (five fish, Figure A1 in Data S1). As a result, the SGR was significantly different between the replicate tanks for mackerel and control treatment, but not for the fish receiving the herring and sprat diets.

**FIGURE 2:**
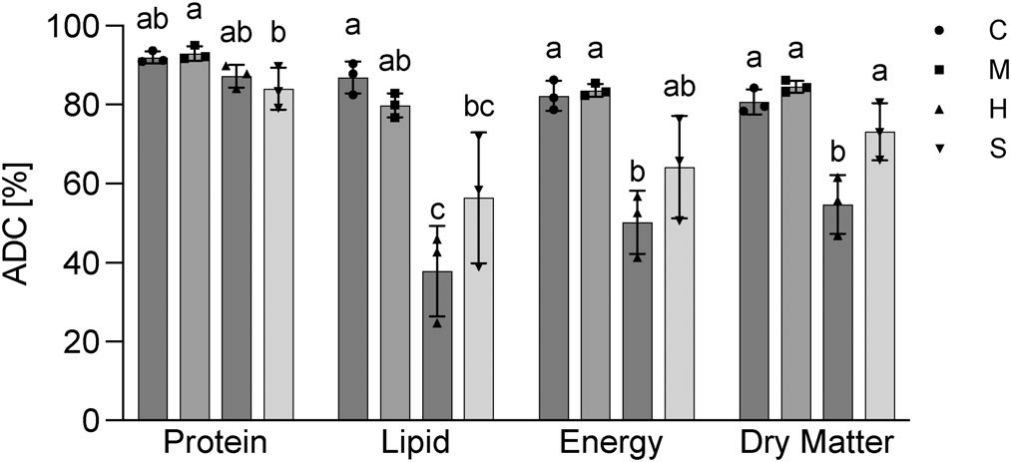
Apparent digestibility coefficient (ADC, %) of crude protein, crude lipid, gross energy and dry matter for the experimental diets: control (C), mackerel (M), herring (H) and sprat (S). Bars indicate mean ± SD. Individual data points are individual replicate tanks. Different letters above bars indicate significant differences (*p* < 0.05) between the groups. Protein, lipid, energy and dry matter were analysed individually.

The ADC for dry matter (DM), crude protein (CP), crude lipid (CL) and gross energy (GE) were numerically highest for the control diet with 80.7 ± 2.6%, 92.0 ± 1.3%, 86.9 ± 3.3% and 82.3 ± 3.1% respectively, Figure 2). Although no significant differences were found between control and mackerel diet, the sprat diet displayed, with 56.4 ± 13.5%, a lower CL ADC compared to the control. The lowest ADC values were observed for the herring diet which displayed lower ADCs than the control for DM (54.6 ± 6.1%), CL (37.83 ± 9.3%) and GE (50.21 ± 6.5%, Figure 2).

### 3.3 Appetite regulation

**FIGURE 3:**
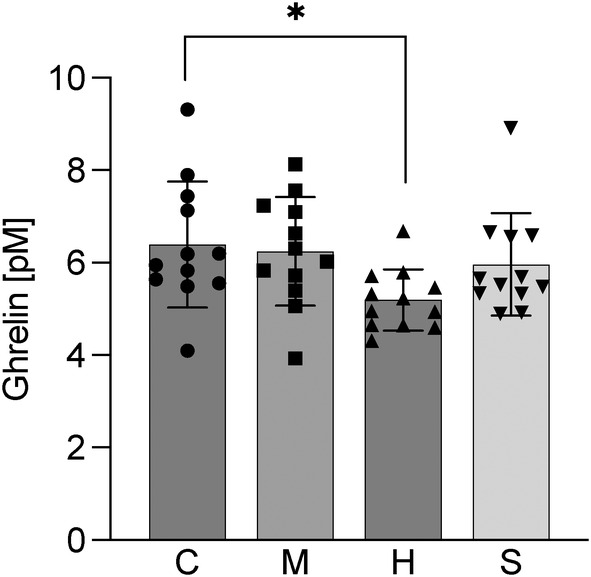
Plasma ghrelin levels of fish fed the four experimental diets, control (C), mackerel (M), herring (H) and sprat (S). Bars indicate mean ± SD. Individual data points are individual fish. The asterisk (*) refers to significant differences between subgroups (*p*‐values <0.05).

The mRNA concentration of all neuropeptides analysed in the hypothalamus was low and no effects of diet on any of the selected appetite‐regulating neuropeptides was observed (Table 5). Plasma ghrelin levels, on the contrary, were significantly lower in the herring diet group (4.8 ± 0.7 pmol/L), compared to the control (6.4 ± 1.3 pmol/L, Figure 3).

**TABLE 5:** Gene expression relative to ubiquitin and β‐Actin mRNA in the hypothalamus for rainbow trout fed one of the experimental diets; control (C), mackerel (M), herring (H) and sprat (S).

	Diet				*p*‐value	
	C	M	H	S	Diet	Tank
CART	32.84 ± 8.76	31.40 ± 6.23	41.47 ± 13.20	30.81 ± 9.50	0.107^b^	
CRF	0.83 ± 0.63	0.76 ± 0.81	0.67 ± 0.89	0.62 ± 0.59	0.863^b^	
MC4R	1.56 ± 0.61	1.65 ± 0.55	1.61 ± 0.45	1.71 ± 0.59	0.759^a^	0.622
NPY	0.94 ± 0.76	1.10 ± 1.37	1.04 ± 1.53	0.73 ± 0.62	0.654^a^	0.009
POMCA1	3.14 ± 3.30	1.40 ± 1.61	6.61 ± 16.60	2.36 ± 3.39	0.516^b^	

*Note*: The data are shown as mean ± S.D., *n* = 4 per tank.

Abbreviations: CART, cocaine‐ and amphetamine‐regulated; CRF, corticotropin‐releasing factor; MC4R, melanocortin 4 receptor; NPY, neuropeptide Y; POMCA1, proopiomelanocortin A1.

^a^ *p*‐values obtained from one‐way ANOVA using log10 transformed data, *n* = 4.

^b^ *p*‐values obtained from a non‐parametric Kruskal‐Wallis ANOVA, *n* = 12.

### 3.4 Stress parameters

**FIGURE 4:**
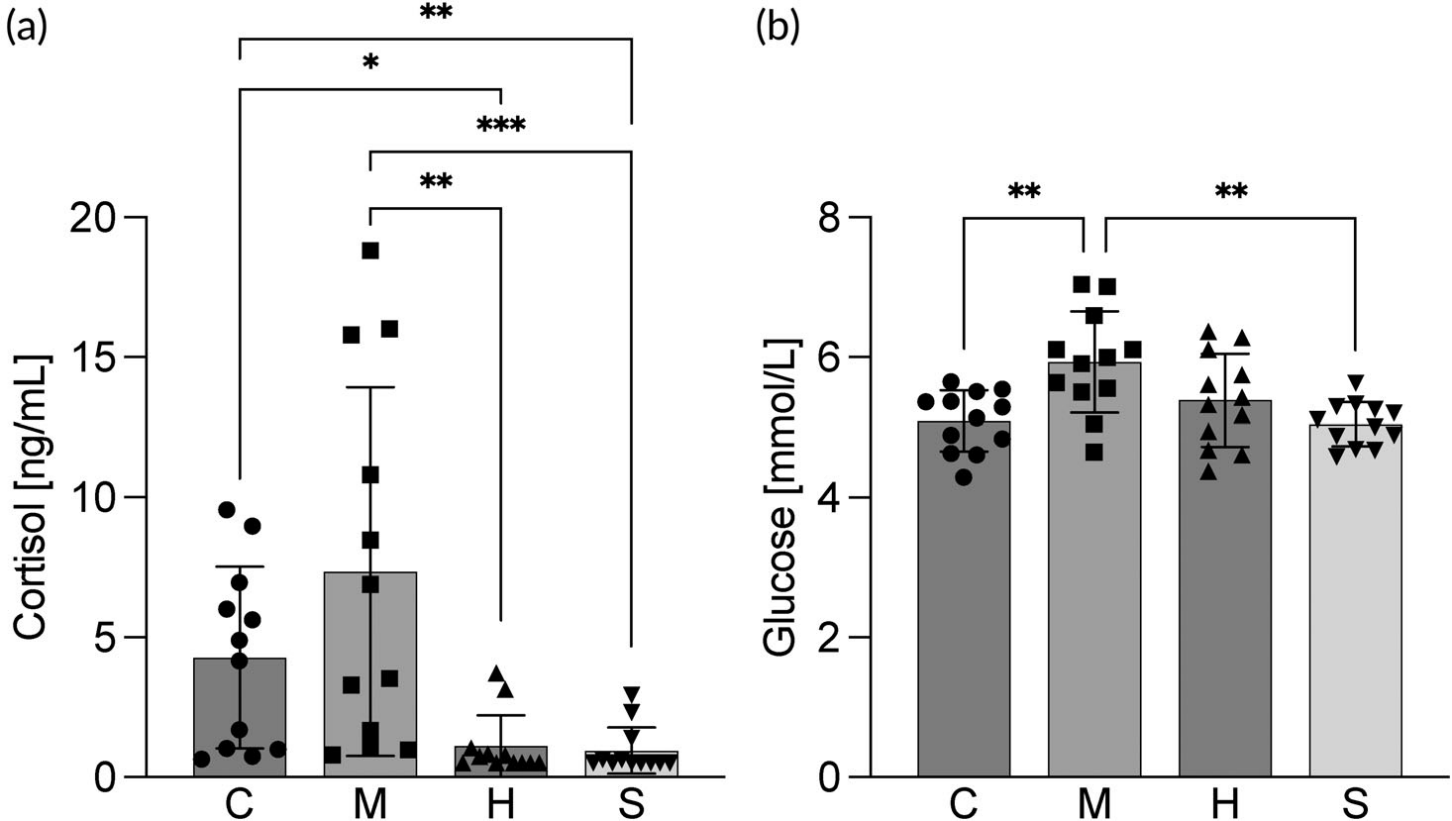
Plasma levels of (a) cortisol and (b) glucose of fish fed the four experimental diets, control (C), mackerel (M), herring (H) and sprat (S). Bars indicate mean ± SD. Individual data points are individual fish. Symbols refer to significant differences between diets, *p* < 0.001 (***); *p* < 0.01(**); and *p* < 0.05 (*).

Plasma cortisol levels were significantly higher for fish in the control (4.3 ± 3.2 ng/mL) and mackerel (7.3 ± 6.6 ng/mL) treatment compared to the herring (1.1 ± 1.1 ng/mL) and sprat (0.8 ± 0.9 ng/mL) treatment (Figure 4). Cortisol levels were also significantly different among the replicated tanks in the mackerel diet (Figure A1 in Data S1). Plasma glucose levels were higher in fish fed the mackerel diet (5.9 ± 0.7 mmol/L) compared to the control (5.1 ± 0.4 mmol/L) and sprat (5.0 ± 0.3 mmol/L) diets.

### 3.5 Intestinal health

#### 3.5.1 Ussing measurements

**FIGURE 5:**
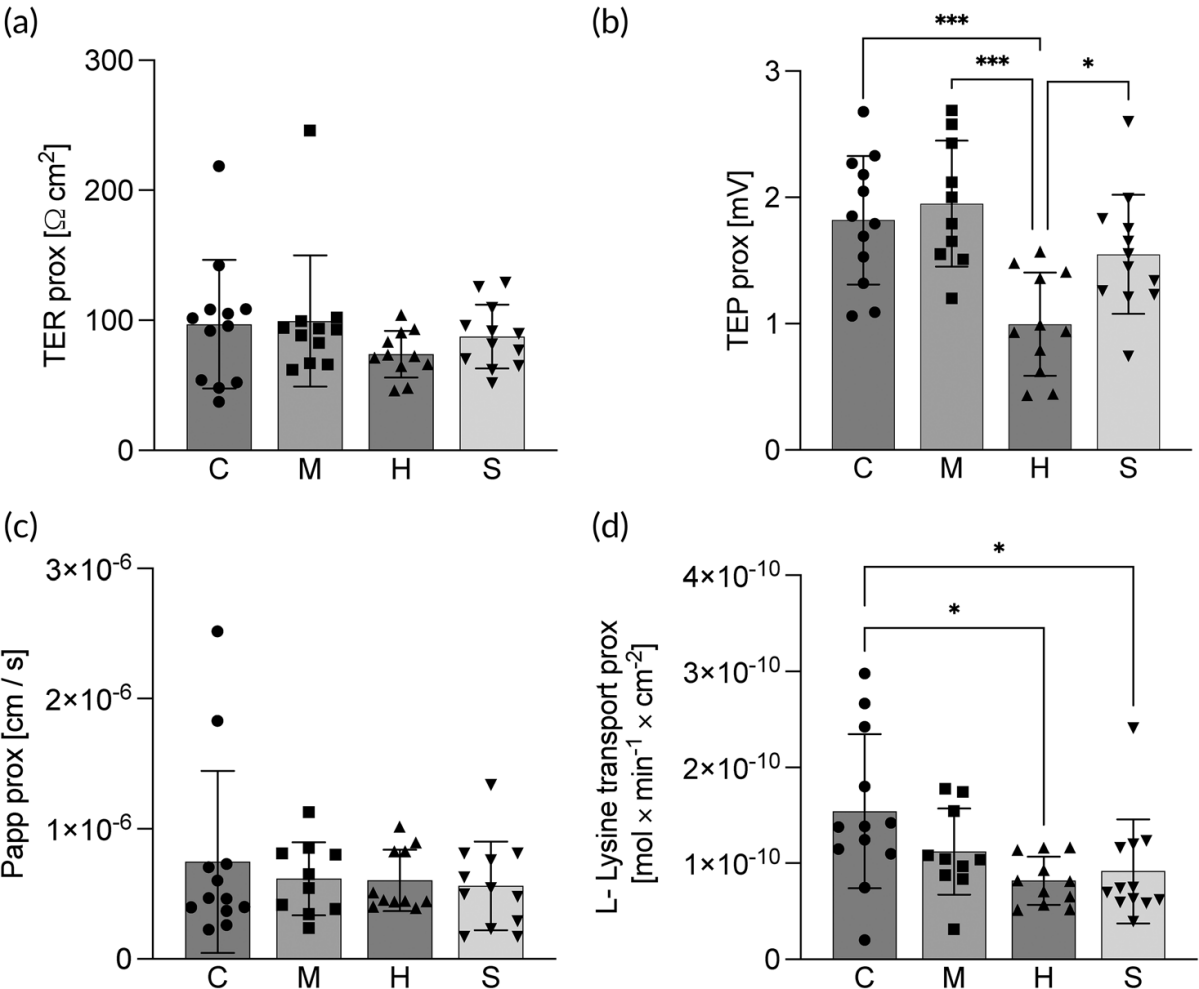
Ussing chamber measurements of (a) transepithelial resistance (TER), (b) transepithelial potential (TEP), (c) permeability for ^14^C‐labeled mannitol (Papp), and (d) uptake rate of ^3^H‐labeled lysine, in the proximal intestine of fish fed the four experimental diets, control (C), mackerel (M), herring (h) and sprat (a). Bars indicate mean ± SD. Individual data points are individual fish. Symbols refer to significant differences between diets, *p* < 0.0001 (****); *p* < 0.001 (***); *p* < 0.01(**); and *p* < 0.05 (*).

The uptake of lysine in the proximal intestine was significantly lower in the herring (0.82 ± 0.3 × 10^10^ mol × min^−1^ × cm^−2^) and sprat (0.92 ± 0.5 × 10^10^ mol × min^−1^ × cm^−2^) treatment compared to the control (1.54 ± 0.8 × 10^10^ mol min^−1^ cm^−2^; Figure 4). The TEP was significantly lower in the fish fed the herring diet (0.99 ± 0.4 mV) compared to those fed mackerel (1.95 ± 0.5 mV), sprat (1.55 ± 0.5 mV) and control diet (1.82 ± 0.5 mV; Figure 5). No differences in any of the measures of intestinal integrity, Papp, TER, and in the SCC were found in the proximal intestine.

**FIGURE 6:**
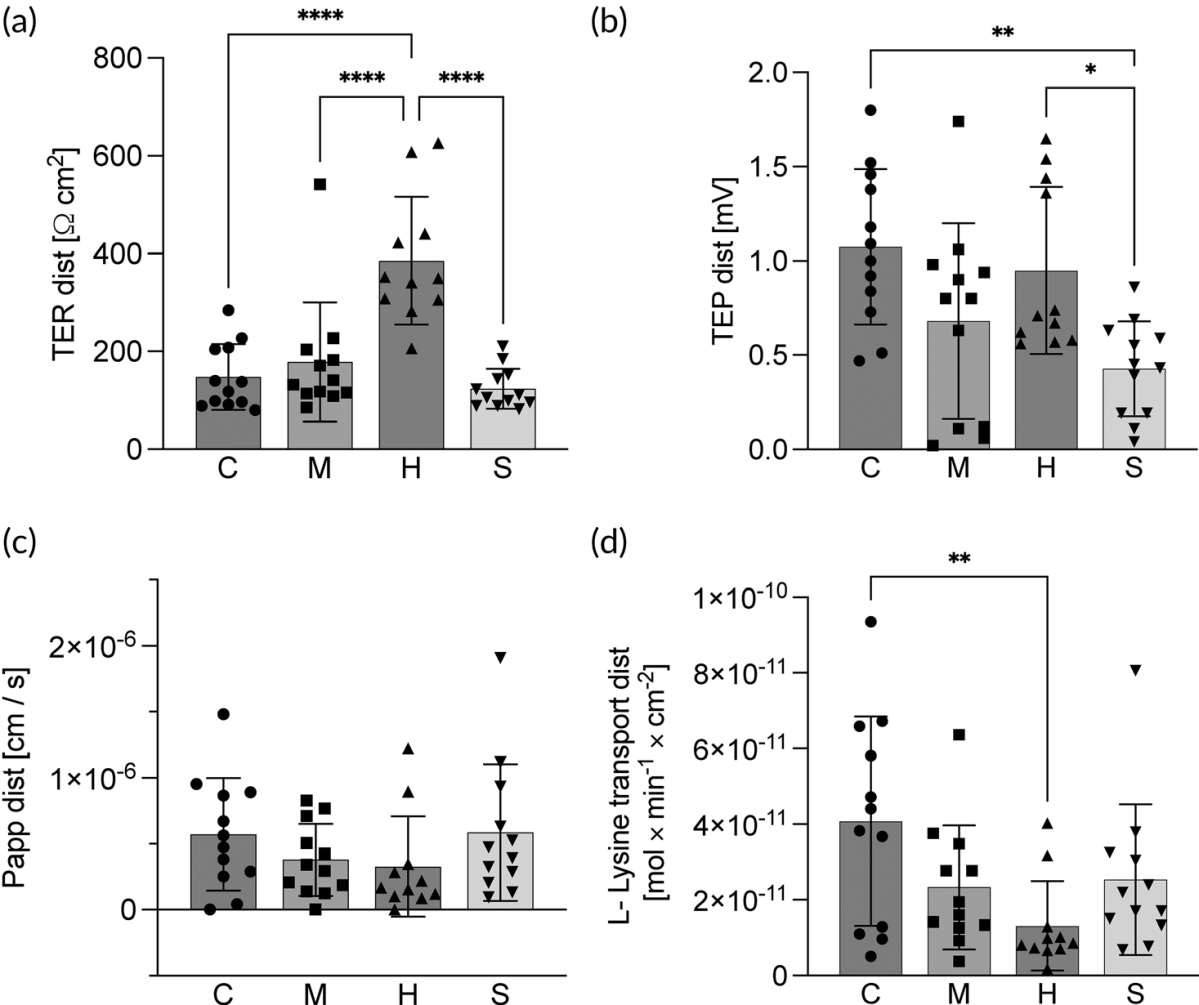
Ussing chamber measurements of (a) transepithelial resistance (TER), (b) transepithelial potential (TEP), (c) permeability for ^14^C‐labeled mannitol (Papp), and (d) uptake rate of ^3^H‐labeled lysine, in the distal intestine of fish fed the four experimental diets, control (C), mackerel (M), herring (h) and sprat (a). Bars indicate mean ± SD. Individual data points are individual fish. Symbols refer to significant differences between diets, *p* < 0.0001 (****); *p* < 0.001 (***); *p* < 0.01(**); and *p* < 0.05 (*).

In the distal intestine of fish fed the herring diet, the TER (386 ± 131 Ω cm^2^) was significantly higher than the TER in the fish fed the other three diets (C: 147 ± 67, M: 178 ± 122 and S: 123 ± 41 Ω cm^2^; Figure 6). No differences between the diets were found in Papp for the distal intestine. As for the proximal intestine, lysine uptake and TEP (4.1 ± 2.8 × 10^11^ mol × min^−1^ × cm^−2^ and 1.1 ± 0.4 mV respectively) were highest for the control with significantly lower levels in lysine uptake of fish fed the herring diet (1.3 ± 1.2 × 10^11^ mol × min^−1^ × cm^−2^), and in TEP in fish fed the sprat diet (0.43 ± 0.25 mV; Figure 6).

#### 3.5.2 Histology

Significant changes in the morphology of both proximal and distal intestines were observed (Table 6). In the proximal intestine, the villi length was higher in fish fed the control (446.7 ± 26.7 μm) and sprat diet (462.3 ± 72.6 μm) compared to the mackerel diet (329.4 ± 82.5 μm), whereas no differences were found for submucosa width, lamina propria width, goblet cell density and vacuolization score.

**TABLE 6:** Histological analysis of proximal and distal intestine of rainbow trout fed one of the experimental diets; control (C), mackerel (M), herring (H) and sprat (S).

	C	M	H	S	*p*‐value
Proximal intestine					
Villi length (μm)	446.7 ± 26.7^a^	329.38 ± 82.5^b^	412.47 ± 43.2^ab^	462.30 ± 72.6^a^	0.005
Submucosa width (μm)	16.03 ± 3.46	15.63 ± 5.22	15.92 ± 3.00	6.49 ± 0.76	0.223^1^
Lamina propria width (μm)	15.36 ± 2.42	12.26 ± 2.16	13.99 ± 1.54	16.51 ± 4.31	0.084
Goblet cell density (/100 μm)	2.23 ± 1.26	2.57 ± 0.93	2.26 ± 0.65	2.29 ± 1.13	0.940
Vacuolization score^2^	1.33 ± 0.53	1.00 ± 0.00	1.00 ± 0.00	1.00 ± 0.00	0.100^1^
Distal intestine					
Villi length (μm)	405.2 ± 62.4	322.4 ± 101.8	372.3 ± 68.4	392.7 ± 95.5	0.359
Submucosa width (μm)	26.21 ± 10.11^a^	11.44 ± 1.10^b^	13.33 ± 2.90^b^	28.85 ± 15.53^a^	0.001^1^
Lamina propria width (μm)	21.00 ± 6.11^a^	13.72 ± 3.9^ab^	10.93 ± 1.92^b^	18.78 ± 6.87^ab^	0.008^1^
Goblet cell density (/100 μm)	2.89 ± 1.22	3.72 ± 0.91	2.57 ± 0.54	2.92 ± 0.69	0.403
Vacuolization score^2^	1.35 ± 0.69	1.08 ± 0.20	1.03 ± 0.07	1.15 ± 0.18	0.444^1^

*Note*: Values within rows with different superscripts are significantly different (*p* < 0.05). The data are shown as mean ± SD, *n* = 6.

^1^ *p*‐values obtained from a non‐parametric Kruskal‐Wallis ANOVA.

^2^ Scoring system adopted from Knudsen et al. (2007): 1 = large vacuoles occupy almost the entire apical part of the enterocytes, 2 = medium‐sized vacuoles, 3 = small‐sized vacuoles are near the apical membrane in most enterocytes, 4 = scattered small vacuoles are still present in some enterocytes and 5 = no supranuclear vacuoles are present.

**FIGURE 7:**
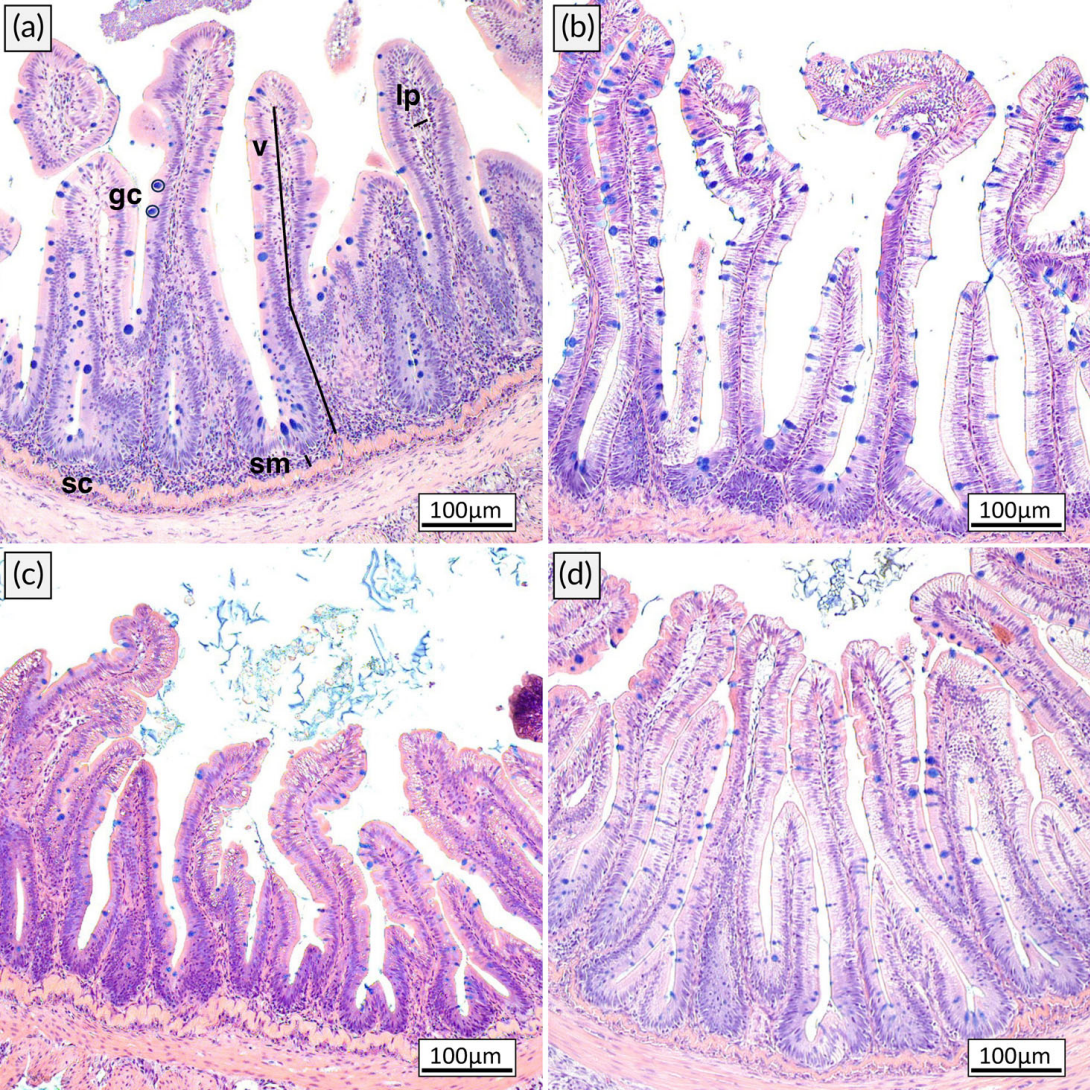
Histological section of the distal intestine of juvenile rainbow trout fed (a) the control, (b) the mackerel, (c) the herring, and (d) the sprat diet. The control and sprat diets resulted in a slightly increased lamina propria and submucosa. Sections were stained using hematoxylin, erythrosine and alcian blue. lp, lamina propria width; sc, stratum compactum; sm, submucosa width; v, villi length and gc; goblet cell count.

In the distal intestine, diet had a significant effect on submucosa and lamina propria width. Fish fed the control and sprat diets exhibited a wider submucosa (C: 26.21 ± 10.11 μm, S: 28.85 ± 15.53 μm) compared to fish fed the mackerel (11.44 ± 1.10 μm) and herring diets (13.33 ± 2.90 μm). The lamina propria width of fish fed the control (21.00 ± 6.11 μm) was significantly higher compared to the herring treatment (10.93 ± 1.92 μm, Table 6, Figure 7).

### 3.6 Hematological parameters

Significant hematological changes were observed in response to the different diets (Table 7). Lower Hb levels were found in the mackerel (6.9 ± 0.9 g/dL) and sprat (6.5 ± 0.5 g/dL) fed fish compared to fish fed the control diet (7.6 ± 0.7 g/dL). Hct levels were significantly affected by diet, as analysed using a nonparametric ANOVA, with the numerically lowest values found in fish fed the sprat diet (30 ± 3%). However, Dunn's pair‐wise comparison yielded no significant results after adjusting for multiple comparisons. MCHC was significantly lower in fish fed the mackerel diet (20.7 ± 1.8 g/dL) compared to the control (22.5 ± 1.3 g/dL) and herring diet (22.6 ± 0.4 g/dL). Analyses of plasma osmolality revealed a higher osmolality in the fish fed herring diet (305.8 ± 5.6 mOsm/kg) compared to the sprat treatment (300.3 ± 4.8 mOsm/kg). Additionally, a tank effect was observed for Hct and MCHC within the mackerel diet, where one tank displayed lower values (Table 7).

**TABLE 7:** Plasma chemistry of rainbow trout fed one of the experimental diets; control (C), mackerel (M), herring (H) and sprat (S).

	Diet				*p*‐value	
	C	M	H	S	Diet	Tank
Hct (%)	33.79 ± 3.32^a^	33.33 ± 5.26^a^	31.21 ± 1.36^ab^	30 ± 3.02^b^	0.025^1^	
Hb (g/dL)	7.58 ± 0.72^a^	6.86 ± 0.94^a^	7.04 ± 0.43^ab^	6.48 ± 0.51^b^	0.001	0.022
MCHC (g/dL)	22.49 ± 1.34^a^	20.71 ± 1.79^b^	22.58 ± 1.35^a^	21.69 ± 1.42^ab^	0.005	0.045
Osmolality (mOsm/kg)	302.0 ± 3.8^ab^	304.2 ± 4.4^ab^	305.8 ± 5.6^a^	300.3 ± 4.8^b^	0.026	0.141
Ghrelin (pmol/L)	6.39 ± 1.36^a^	6.24 ± 1.17^ab^	4.77 ± 0.66^b^	5.96 ± 1.11^ab^	0.028	0.052

*Note*: The data are shown as mean ± SD, *n* = 4 per tank. Values within rows with different superscripts are significantly different (*p* < 0.05).

Abbreviations: Hb, hemoglobin; Hct, hematocrit; MCHC, mean corpuscular hemoglobin concentration.

^1^ *p*‐values obtained from a nonparametric Kruskal‐Wallis ANOVA.

## 4 DISCUSSION

Food production side streams are increasingly being recognized as valuable resources rather than waste products (Stevens et al., 2018). However, side streams are highly diverse and much of their potential as food or feed sources is still untapped (Sandström et al., 2022). Here we evaluated three locally produced side streams for their potential as feed ingredients, without additional processing. The first objective in this assessment was a screening of the chemical composition of the three test ingredients. The sprat trimmings represented primary fileting side streams, whereas both the herring and mackerel consisted of fillets, further down the production line. As a result, the protein content of the herring and mackerel fillets was higher than that of the sprat trimmings. Compared to pure fillets of the same species, the protein content of the herring and mackerel material was slightly lower probably due to a combination of added ingredients (tomato sauce and brine) and nutrient leaching into processing water, brine and sauce (Bazarnova et al., 2020; Gall et al., 1983; USDA, 2018). The protein content of the sprat trimmings was in line with similar filleting side streams (Bechtel, 2003; Hinchcliffe et al., 2019). The lipid content of all three raw materials was above 30%. Both herring and mackerel are “fatty fish” with fillet lipid contents naturally between 20%–30% (Aidos et al., 2002; Bastías et al., 2017). While important in a balanced fish feed, high lipid contents in the final mash (above ca. 6% depending on extruder) can reduce extrusion shear and pellet expansion (Camire & Krumhar, 1990; Singh et al., 2007). To reach the high lipid (ca. 30%) content desired for high energy salmonid grow‐out diets, feed pellets are vacuum coated after the extrusion. The high lipid content of the three tested side streams may therefore restrict their total inclusion level in extruded diets. For the commercial application of these side streams, a separation of the lipid and protein fractions could improve not only the degree of utilization in feed formulations but also the storage time, as high lipid levels are more rapidly oxidized. That these steps require additional processing and therefore generate additional costs should be considered (Ween et al., 2017). In the present study, all diets were produced via cold pelleting to be able to include as much as 50% of the three test ingredients.

Ash (minerals and salts) and phosphate levels were highest in the sprat trimmings (26% and 1.4% respectively) due to the presence of bone and cartilage, as well as the addition of salt as a preservative (Toppe et al., 2007). When used in fish feed, an ingredient with a high ash content can “dilute” the feeds' nutrient content, which needs to be considered when formulating high energy diets. A lower nutrient content in the final diet may in turn increase the FCR and likely the environmental footprint of the farming operation. The high ash content of the raw material resulted in 16% ash in the sprat diet. However, if not containing elevated levels of certain metals, such high ash levels may not negatively affect growth if feed is provided albidum in salmonid fish (Shearer et al., 1992). As fishmeal produced from filleting side streams commonly contains above 20% ash, the ash content of the sprat trimmings needs be considered in the feed formulations but should not hinder commercial applications (Bechtel, 2003; Ween et al., 2017). Phosphorous is an essential mineral, needed for the formation of for example bones, scales and various biomolecules including ATP and RNA/DNA. Therefore, phosphorous is essential in a balanced fish feed. At the same time, phosphorous is a limiting factor, especially in freshwater ecosystems, and a prime cause of eutrophication (Luo, 2022). In aquaculture systems, phosphorous therefore needs to be managed responsibly, meaning that phosphorous levels in the feed need to be tailored to reduce leakage while satisfying the nutritional needs of the fish. This is especially true in light of looming shortages in the global phosphorous supply (Desmidt et al., 2015). The high phosphorous value, in the sprat trimmings may therefore be an added value but excess levels in the feed should be avoided.

In summary, all three side streams contained sufficient amounts of protein and lipid. As herring and sprat are commonly used to produce fishmeal, this was expected. The sprat trimmings additionally contained high levels of ash and phosphorous, which can add value but needs to be considered in feed formulations to avoid negative environmental and physiological effects.

The second objective of the present study was to evaluate possible effects of the side streams on appetite, digestibility, and feed utilization when included in diets for rainbow trout. Fish fed the sprat diet displayed the highest feed intake and SGR. The sprat trimmings underwent the least processing, as only salt was added for preservation, and were the freshest of the three side streams as the filleting trimmings were extracted shortly after the fish were caught. Therefore, the sprat trimmings likely maintained a “natural” taste. Compared to the control diet, the lipid digestibility of the sprat diet was low, likely due to the high amount of cartilage and bone in the feed which is supported by a previous study where the inclusion of fish bone meal resulted in a lower lipid and energy ADC in rainbow trout (Lee et al., 2010).

Fish fed the mackerel diet displayed a lower SGR and feed intake compared the sprat. The mackerel side stream was subjected to the highest degree of processing, altering both chemical composition and taste through the addition of seasoning and tomato sauce. However, due to the addition of antioxidants in the tomato sauce as part of the food production process, the mackerel fillets likely remained fresh. There was no difference between control and mackerel diet regarding growth and feed intake. As these processing steps probably affected taste and nutrient composition strongly, the observed feed intake and growth rates suggest the mackerel side stream to be a promising feed ingredient. Additionally, the mackerel displayed the highest digestibility of the three test ingredients. Both the mackerel and control diets showed digestibility values commonly observed for pelleted diets in rainbow trout (Cheng & Hardy, 2003).

Fish fed the herring diet displayed the lowest feed intake which was concomitant with a reduction in weight gain and SGR. These results suggest that feed intake is a main driver for the observed difference in growth. Coinciding with a reduced feed intake, fish fed the herring diet exhibited decreased plasma ghrelin levels. In mammals and several fish, ghrelin is the only known peripheral orexigenic (feed‐stimulating) hormone and is directly linked to the release of growth hormone and somatic growth (Kojima et al., 1999; Nakazato et al., 2001; Rønnestad et al., 2017). However, its role in rainbow trout is more ambiguous as both anorexigenic and orexigenic functions have been observed (Jönsson et al., 2010; Velasco et al., 2016). The present results are in line with the latter, and would support an orexigenic function, as rainbow trout fed the herring diet had the lowest ghrelin levels along with a lower feed intake and growth. The two central systems governing food intake; the homeostatic, controlling energy balance, and the hedonic, motivated by pleasure and reward, act in a complementary fashion (Lutter & Nestler, 2009). In the present experiment, feed was provided in abundance to all fish, likely increasing the relative importance of hedonic feeding habits. In rainbow trout both hedonic and homeostatic pathways have been identified, implying the existence of substances that stimulate or inhibit feed intake beyond their nutritional value (Díaz‐rúa et al., 2020a; Rønnestad et al., 2017). Indeed, various feed intake stimulating and deterring substances have been identified for rainbow trout (Bureau et al., 1998; Díaz‐Rúa et al., 2020b; Yamashita et al., 2006). One class of deterrents are lipid peroxidation products that have been shown to decrease feed intake in Atlantic salmon (*Salmo salar*) and rainbow trout (Hamre et al., 2001; Lund et al., 2013; Sutton et al., 2006). The herring product was the least “fresh” of the three raw materials tested due to the extended storage and processing. Although not explicitly measured the herring product likely displayed the most advanced lipid peroxidation of the three side streams (Wu et al., 2022). Interestingly, peroxidation products have also been shown to reduce digestibility and feed utilization in rainbow trout (Forster et al., 1988; Lund et al., 2013). In concordance, fish fed the herring diet displayed the lowest digestibility values and a lipid ADC of below 50%. The low lipid ADC likely led to an increased number of amino acids utilized for energy rather than growth. This effect may be amplified by the fact that the experimental diets contained *ca* 10% lower lipid levels compared to commercial trout feeds. Additionally, the low lipid ADC suggests considerable amounts of lipid leakage which may affect the environment negatively. Thus, the lowered appetite, palatability, and digestibility likely all contributed to the lower growth of fish fed the herring diet.

The growth rates in the groups fed control and mackerel diets were less homogenous compared to the sprat and herring diets and significant differences were observed between the replicate tanks. At the same time, cortisol levels in the control and mackerel diets, as well as plasma glucose in the mackerel diet, were higher than those in sprat and herring diets, possibly indicating mild stress (Barton et al., 1986; Bry, 1982). These observations may indicate that hierarchical structures were developed in certain tanks resulting in mild social stress in some individuals and a discrepancy in feed intake and growth between the dominant and subordinate fish (Gregory & Wood, 1999; Sloman et al., 2001). Stocking densities below 10 kg/m^3^ have been shown to contribute to hierarchical and territorial behavior among rainbow trout (North et al., 2006). However, in the present study, the lowest density at the start of the experiment was 11 kg/m^3^ which rose to 27 kg/m^3^ at the end. Thus, the densities were kept within the recommended range throughout the experiment. Even though the experimental setup including the feeding regime, with controlled hand‐feeding twice daily, was designed to avoid hierarchical structures, these may arise regardless, due to individual differences in behavior among fish (Noakes & Leatherland, 1977). Nonetheless, average cortisol levels of fish from both the control and mackerel groups were below 10 ng/mL, corresponding to no or very low stress levels (Bry, 1982; Sloman et al., 2001).

In summary, fish fed the herring diet displayed the lowest performance regarding growth, feed intake and digestibility. The mackerel side stream was more promising due to good digestibility values as well as growth rates not significantly lower than the control. The sprat diet had a positive effect on feed intake and performed well in terms of growth while the high bone content may reduce digestibility. Despite the tank effect for control and mackerel diets, the general effects of diet with high performance for sprat, medium performance for mackerel and low performance for the herring diet were apparent for many of the analysed parameters.

The third objective of the present study was to evaluate possible effects of the side streams on health and welfare with a special focus on the intestinal physiology when included in diets for rainbow trout. As for the growth and feed utilization, no difference was observed between the control and mackerel treatment regarding the intestinal barrier function. In line with the reduced feed intake and growth, the herring diet had a pronounced negative effect on the intestinal physiology. Despite the high growth performance, slight physiological changes were also observed for the fish fed the sprat diet. Lysine uptake in the proximal intestine of fish fed the sprat diet was lower compared to the control. A lower lysine uptake rate may be the result of a decrease in the epithelial surface area as is commonly observed for fish with enteritis (Knudsen et al., 2008; Krogdahl et al., 2010). However, the histological analysis revealed no indication for an enteritis in the proximal intestine of fish fed the sprat diet. Considering the high growth rate and low feed conversion ratio, the lower lysine uptake observed in the in vitro measurements may not directly reflect the situation at the whole animal level. In vivo, chyme transient time, nutrient and minerals in the feed may all contribute to a sufficient nutrient uptake. The herring diet on the contrary, had a clear negative effect on intestinal health with a lower lysine uptake compared to the control in both proximal and distal intestine. As fish fed the herring diet did not show alterations in the intestinal histology, the reduced lysine uptake may be related to decreased epithelial function on a cellular level that is supported by lower TEP in the proximal intestine compared to the control and sprat treatments. The TEP reflects the potential difference generated by the passive transfer and active transport of charged molecules across the epithelium. In the proximal intestine of rainbow trout, most of the amino acid uptake, including lysine, is sodium dependent as Na^+^/amino acid symporters rely on the sodium gradient created by the enterocytes' Na^+^K^+^‐ATPases (Hedén et al., 2022; Sundell & Sundh, 2012). The lower serosa positive TEP may therefore be a consequence of a reduction in Na^+^ driven nutrient (including lysine) transport. This is further supported by the maintained TER indicating no differences in passive paracellular nutrient uptake in this intestinal region. In the distal intestine, on the contrary, amino acid uptake is overall much lower and more reliant on less specific processes such as endocytosis and paracellular diffusion (Grosell et al., 2010; Günzel & Yu, 2013; Karasov, 2017). The lower lysine uptake of herring fed fish in this region may therefore be a consequence of the higher TER compared to the control fish, reflecting lower paracellular permeability. The lower lysine uptake in both proximal and distal intestines may partly reflect the low digestibility of fish fed the herring diet and thus contribute to the observed lower growth in this group.

The histological analysis revealed a wider lamina propria and submucosa in the distal intestine of fish fed the sprat and control diets. The lamina propria and submucosa widths, together with the villi length, goblet cell density, and the vacuolization score, are collectively considered histopathological indicators for inflammation (Baeverfjord & Krogdahl, 1996). However, changes in only one or few of these indicators may be less conclusive. In this study, no changes were observed for the other parameters, villi length, vacuolization score, and goblet cell count in the distal intestine. Furthermore, the increase in the lamina propria and submucosa width was small compared to previous studies where an inflammation was observed (Baeverfjord & Krogdahl, 1996; Knudsen et al., 2007; Krogdahl et al., 2010).

Small hematological differences were observed between the treatment groups. Considering the high inclusion level, 50%, of three heterogeneous ingredients, variations in the hematological profile can be expected. However, the differences were small between the dietary groups and all parameters were within the normal range for rainbow trout indicating overall good health (Barnhart, 1969; Blaxhall & Daisley, 1973; Greene & Selivonchick, 1990; McCarthy et al., 1973).

In summary, both the mackerel and sprat diets are promising feed ingredients considering intestinal physiology and health. Fish fed the mackerel diet displayed no negative effects on intestinal physiological and overall health. The fish fed the sprat diet showed a slightly lower lysine uptake, which, however, did not reduce protein utilization in the whole animal. Fish fed the herring diet, displayed a reduced lysine in the intestine, which is in line with the overall lower growth and digestibility in this group.

## 5 CONCLUSION

Fish processing side streams can be valuable raw materials and may be especially suited as feed ingredients for carnivorous fish. The primary side stream, sprat trimmings, as well as the secondary side stream, marinated mackerel fillets, have high potential as alternative ingredients in diets for rainbow trout. The sprat trimmings were the freshest and yielded the highest growth rates and feed intake making it the most promising side stream. However, the maximum inclusion level of this side stream in diets for salmonid fish may be limited by the high lipid and ash content. The mackerel side stream may be less promising than the sprat trimmings due to the observed lower growth and feed intake. However due to a high digestibility and growth rates similar to the control diet, the mackerel side stream is still worth further investigation but maybe need additional processing and/or require even lower inclusion levels. The secondary side stream, marinated herring, on the contrary, will require additional processing to be considered as a feed ingredient. The herring diet resulted in reduced growth likely due to a combination of negative effects on feed intake, appetite, digestibility, and intestinal barrier function, which may be the result of a lower freshness and higher degree of lipid peroxidation of this side stream. All three side streams contained high amounts of lipids. To improve storage and transport, a separation of protein and lipid fractions would be beneficial. However, for feed producers in close proximity to the seafood processing industry, the sprat trimmings and mackerel in tomato sauce can potentially be used directly. This would increase the freshness, decrease the need for additional processing, and thereby reduce costs and likely the environmental footprint. As part of a more local and circular food production, in line with the 2030 Agenda for Sustainable Development, set out by the United Nations, retaining side streams in the food production chain is highly encouraged. The two side streams evaluated in the current study are especially promising in light of the increasing demand and finite supply of fishmeal and fish oil.

## AUTHOR CONTRIBUTION

Niklas Warwas: conceptualization, funding acquisition, methodology, formal analysis, investigation, visualization, writing—original draft. Markus Langeland: conceptualization, supervision, funding acquisition, writing—review and editing. Jonathan A.C. Roques: investigation, methodology, writing—review and editing. Marie Montjouridès: investigation. Jolie Smeets: formal analysis, investigation. Henrik Sundh: supervision, writing—review and editing. Elisabeth Jönsson: supervision, methodology, writing—review and editing. Kristina Sundell: conceptualization, methodology, supervision, funding acquisition, project administration, writing—review and editing.

## FUNDING INFORMATION

We would like to thank the Swedish Board of Agriculture (Dnr: 3.3.17‐17137/2020), The Royal Swedish Academy of Agriculture and Forestry (KSLA, GFS2021‐0040), the Swedish Mariculture Research Center (SWEMARC) and the Blue Food – Center for the Seafood of the Future (FORMAS, Dnr: 2020–02834) for financing this project.

## CONFLICT OF INTEREST STATEMENT

The authors have no conflicts of interest to declare.

## Supporting information


**Data S1.** Supporting Information.

## Data Availability

The data that support the findings of this study are available from the corresponding author upon reasonable request.
