# Supplementary material for: Fish processing side streams are promising ingredients in diets for rainbow trout (Oncorhynchus mykiss) —Effects on growth physiology, appetite, and intestinal health
Source: J Fish Biol. 2023 Oct 30;106(1):75–92. doi: 10.1111/jfb.15589 (PMC11758193; doi:10.1111/jfb.15589)
Supplement: Supplementary file 1 — Data S1. Supporting Information. [file JFB-106-75-s001.docx]

Supplement

| Table A1  Chemical composition (g/100g DM) of raw materials: mackerel in tomato sauce (M), marinated herring (H) and sprat trimmings (S). The ingredients underwent additional washing before the feed formulation. | | | | | |
| --- | --- | --- | --- | --- | --- |
|  | Mackerel | Herring | Sprat | Fishmeal^3^ | Requirements^4^ |
| *Proximate composition* |  |  |  |  |  |
| Ash | 4.4 | 25.2 | 26.2 | 13.9 |  |
| Crude protein^1^ | 34.5 | 30.9 | 26.0 | 67 |  |
| Sum amino acids | 28.8 | 34.4 | 21.4 | 62 |  |
| Crude lipids^2^ | 54.7 | 42.4 | 40.5 | 10.9 |  |
| Neutral detergent fiber | 5.9 | 1.7 | 7.4 | - |  |
|  |  |  |  |  |  |
| *Amino acids* |  |  |  |  |  |
| Alanine | 1.8 | 2.1 | 1.4 | 4.1 |  |
| Arginine | 1.7 | 2.2 | 1.3 | 3.9 | 1.38 |
| Aspartic acid | 3.1 | 3.7 | 2.1 | 5.8 |  |
| Glutamic acids | 4.6 | 5.2 | 2.9 | 8.3 |  |
| Glycine | 1.8 | 1.6 | 1.5 | 4.3 |  |
| Histidine | 1.3 | 0.9 | 0.7 | 2.5 | 0.85 |
| Isoleucine | 1.2 | 1.7 | 1.0 | 3 | 1.12 |
| Leucine | 2.2 | 3.0 | 1.7 | 5 | 2.02 |
| Lysine | 2.5 | 3.3 | 1.8 | 5.3 | 2.06 |
| Phenylalanine | 1.1 | 1.5 | 1.0 | 2.7 | 1.28 |
| Proline | 1.2 | 1.2 | 0.9 | 2.7 |  |
| Serine | 1.3 | 1.5 | 1.0 | 2.2 |  |
| Threonine | 1.4 | 1.7 | 1.0 | 2.5 | 1.12 |
| Tyrosine | 1.0 | 1.3 | 0.8 | 2.4 |  |
| Valine | 1.5 | 2.0 | 1.2 | 3.7 | 1.43 |
| Cysteine + Cystine | 0.2 | 0.3 | 0.2 | 0.9 |  |
| Methionine | 0.6 | 0.8 | 0.6 | 2 | 0.82 |
| Tryptophan | 0.4 | 0.5 | 0.3 |  | 0.24 |
|  |  |  |  |  |  |
| *Fatty acids* |  |  |  |  |  |
| C 16:0 | 5.6 | 5.2 | 7.0 | 1.31 |  |
| C 16:1 n-7 | 1.6 | 1.9 | 2.6 | 0.38 |  |
| C 18:1 | 8.5 | 3.5 | 6.0 | 0.6 |  |
| C 18:2 n-6 | 2.0 | 0.6 | 0.6 | 0.06 |  |
| C 18:3 n-3 | 1.3 | 0.5 | 0.6 | 0.05 |  |
| C 18:4 n-3 | 2.5 | 1.6 | 1.1 | 0.14 |  |
| C 20:1 n-9 | 5.3 | 4.7 | 1.5 | 0.06 |  |
| C 20:5 n-3 (EPA) | 3.3 | 2.9 | 3.5 | 0.95 |  |
| C 22:1 | 9.0 | 9.5 | 3.4 | 0.04 |  |
| C 22:6 n-3 (DHA) | 5.2 | 3.7 | 5.5 | 1.46 |  |
| Total saturated fatty acids | 10.2 | 9.1 | 11.2 | 2.02 |  |
| Total single unsaturated fatty acids | 24.9 | 20.0 | 13.9 | 1.35 |  |
| Total polyunsaturated fatty acids | 16.0 | 10.1 | 12.4 | 0.48 |  |
| Total omega 6 fatty acids | 2.5 | 0.9 | 1.1 | 0.19 |  |
| Total omega 3 fatty acids | 13.5 | 9.3 | 11.4 | 1.35 |  |
| Ratio omega6/omega3 fatty acids | 0.2 | <0.10 | <0.10 | <0.10 |  |
| Phosphorous (g/100g) | 0.34 | 0.19 | 0.88 | <0.01 |  |
| ^1^According to Kjeldahl (N*6.25)  ^2^According to Schmid-Bondzynski-Ratslaff  ^3^TASA, Anchoveta, Peru, Glencross, 2020, A feed is still only as good as its ingredients: An update on the nutritional research strategies for the optimal evaluation of ingredients for aquaculture feeds  ^4^Amino acid requriments according to Hua & Bureau, 2019. | | | | | |

Figure A1. Specific growth rate (SGR, a) and plasma cortisol (b) of rainbow trout fed 4 different experimental diets, control (C), mackerel (M), herring (H) and sprat (S). Bars indicate mean ± SD. Individual data point are individual fish. Symbols refer to significant differences between replicate tanks, *p* <0.0001 (****), <0.001 (***), <0.01(**), and <0.05 (*). Different letters above bars indicate significant main effects between treatments *p* < 0.05.
